# Prognostic Value of Characterizing Myocardial Tissue by Cardiac MRI with T1 Mapping in HFpEF Patients: A Systematic Review and Meta-Analysis

**DOI:** 10.3390/jcm11092531

**Published:** 2022-04-30

**Authors:** Elena Golukhova, Naida Bulaeva, Svetlana Alexandrova, Olga Gromova, Bektur Berdibekov

**Affiliations:** Bakulev Scientific Center for Cardiovascular Surgery, 121552 Moscow, Russia; egolukhova@yahoo.com (E.G.); naida_bulaeva@yahoo.com (N.B.); svaleksandrova@yandex.ru (S.A.); gromova3112@gmail.com (O.G.)

**Keywords:** cardiac MRI, HFpEF, T1 mapping, extracellular volume, native T1, postcontrast T1, prognosis

## Abstract

Objectives: Our study aimed at conducting a systematic review and meta-analysis, with the objective of evaluating the prognostic value of T1 mapping techniques via cardiac magnetic resonance (CMR) in heart failure with preserved ejection fraction (HFpEF) patients. Materials and methods: The protocol was prospectively registered in the international prospective register of systematic reviews PROSPERO (registration number CRD42022300991). We searched PubMed, Google Scholar, and EMBASE for studies examining the prognostic value of characterizing myocardial tissue via CMR imaging with T1 mapping in HFpEF. Hazard ratios (HRs) for uniformly defined predictors were pooled for meta-analysis. Results: In total, 7 studies were retrieved from 351 publications for this systematic review and meta-analysis. A total of 1930 patients (mean age of 69.4 years, mean follow-up duration of 25.6 months) was included in the analysis. The meta-analysis demonstrated that higher extracellular volume (ECV) was associated with an increased risk of death and/or hospitalization with heart failure (HF) (HR:1.12; 95% CI: 1.06–1.18; *p* < 0.0001). After adjusting for baseline characteristics, the higher extent of ECV remained strongly associated with the risk of death and/or hospitalization with HF (HR_adjusted_: 1.08; 95% CI: 1.04–1.13; *p* = 0.0001). However, no significant association of native T1 value with risk of death or adverse cardiovascular events was found (HR:1.01; 95% CI: 1.00–1.02; *p* = 0.21). Conclusion: Assessment of ECV via CMR has an important prognostic value for outcomes of death and/or hospitalization with HF, and can therefore be used as an effective tool for risk stratification of patients with HFpEF.

## 1. Introduction

Heart failure with preserved ejection fraction (HFpEF) is a clinical syndrome of patients with symptoms and signs of heart failure (HF) with normal or near-normal left ventricular ejection fraction (LVEF ≥ 50%). The prevalence of patients with HFpEF is progressively increasing, accounting for over 50% of all HF cases [1]. Data from several studies suggest that focal or diffuse fibrosis is involved in the pathophysiology of HFpEF [2]. CMR imaging has become increasingly available, and is well established in the assessment of cardiac morphology and function. Late gadolinium enhancement (LGE) and T1 mapping are valuable CMR tools for the detection of myocardial fibrosis, infiltration, and scarring. While LGE can solely detect the focal myocardial fibrosis, T1 mapping is a novel CMR-based technique for myocardial tissue characterization, capable of identifying diffuse fibrosis [3]. Several T1 mapping techniques have been used in published studies, including postcontrast T1 mapping, calculation of extracellular volume fraction via MOLLI sequences, and native T1 mapping. However, at present, there is no consensus regarding the most accurate mapping approach, and the role of various mapping techniques in predicting and assessing the outcomes in HFpEF patients has not been properly evaluated [4,5].

The objective of this systematic review and meta-analysis was to evaluate the prognostic value of novel T1 mapping indices in HFpEF patients, viz., native T1, postcontrast T1, and ECV.

## 2. Materials and Methods

This study was conducted in accordance with the Preferred Reporting Items for Systematic Reviews and Meta-Analyses (PRISMA) [6]. The protocol was registered prospectively in PROSPERO—an international prospective register of systematic reviews (registration number CRD42022300991). 

### 2.1. Eligibility Criteria 

The criteria for inclusion in this meta-analysis were studies that (1) included a cohort of participants with HFpEF, (2) performed CMR T1 mapping (i.e., native T1, postcontrast T1, or ECV), (3) reported predictors (i.e., native T1, postcontrast T1, or ECV) of clinical outcomes in HFpEF patients obtained through univariate and/or multivariate analyses, and (4) had a follow-up period of over 6 months. The exclusion criteria were as follows: (1) articles dealing with non-human subjects; (2) articles written in languages other than English.

### 2.2. Literature Searching Strategy 

We systematically searched PubMed, EMBASE, and Google Scholar all the way through January 2022, using the following keywords: ((T1 mapping) OR (native T1) OR (postcontrast T1) OR (extracellular volume fraction) OR (extracellular matrix)) AND ((outcome)) and ((prognosis)) AND ((heart failure with preserved ejection fraction) OR (HFpEF)). Data mining and analyses were performed independently by two researchers (B.B. and N.B.). Any disagreements between regarding the eligibility of particular studies were resolved via discussion with a third reviewer (O.G.). 

### 2.3. Data Mining and Synthesis 

Data were independently extracted into a prespecified data extraction table. The primary outcomes of interest were hospitalization for HF and all-cause mortality. The Newcastle–Ottawa Score (NOS) for observational studies was used for assessing the risk of bias [7,8]. The following characteristics were assessed: (1) representativeness of the exposed cohort; (2) selection of the unexposed cohort; (3) establishment of exposure; (4) demonstrating the absence of the outcome of interest at baseline; (5) comparability of cohorts based on study design or analysis; (6) evaluation of outcomes; (7) follow-up periods long enough for outcomes to take place; and (8) the adequacy of cohort follow-up. Each study was assigned a score from 0 to 9. Depending on their score, the studies were considered to be of a low quality (<5), medium quality (5–7), or high quality (>7). We only included studies of medium or high quality.

### 2.4. Statistical Analysis

The meta-analysis was carried out by applying the conventional statistical analysis models using RevMan (Review Manager) version 5.1 (the Cochrane Collaboration, Copenhagen, Denmark) and Comprehensive Meta-Analysis 3.0 (Biostat, Englewood, NJ, USA). The I2 statistic was employed to assess heterogeneity between studies, with I^2^ values of 25%, 50%, and 75% representing low, moderate, and high heterogeneity, respectively. We pooled together the values of HR for clinical outcomes and the number of patients recruited in each study. Inverse variance weighting was used to calculate the total hazard ratios (HRs) within a random-effects model. Heterogeneity statistics were included to calculate the overall correlation coefficient within the 95% confidence interval (CI) random-effects model that was used in all analyses. The main results of the meta-analysis were presented in the form of forest plots. Publication bias was evaluated using Egger’s regression test.

## 3. Results

### 3.1. Results of Literature Search 

**Figure 1:**
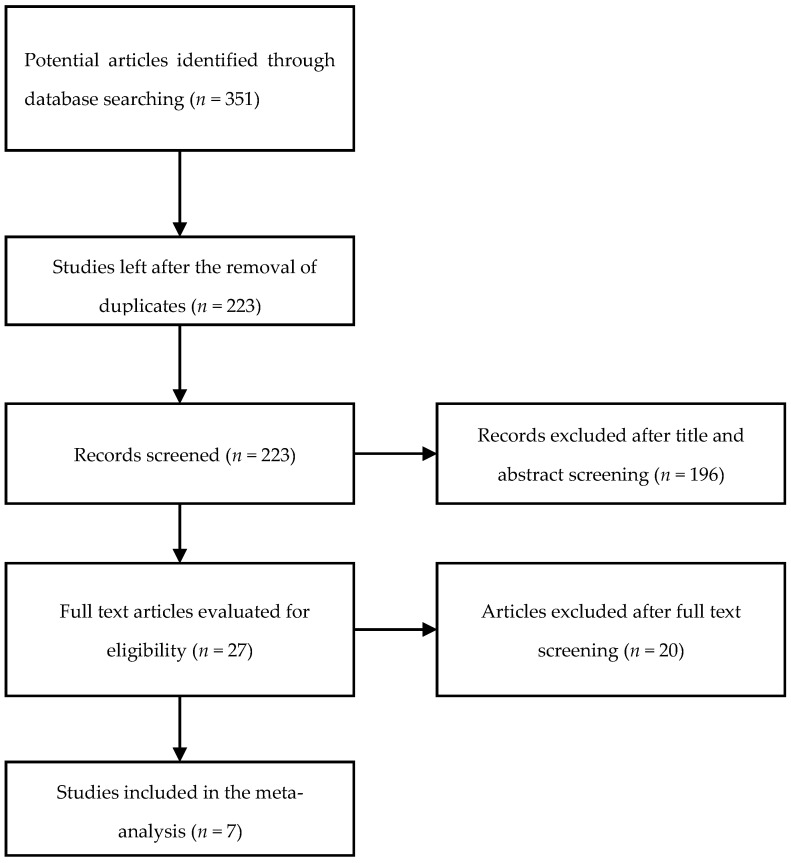
Study selection flowchart.

A total of 351 publications was identified through our search of the PubMed, EMBASE, and Google Scholar databases. Following the screening of the titles and abstracts, 27 articles were selected for the full-text review. Finally, after excluding articles not meeting the inclusion criteria, as well as duplicate studies, seven studies were identified and included in the systematic review and meta-analysis (Figure 1).

### 3.2. Baseline Characteristics of the Studies

The total number of included patients was 1930, and the mean duration of the follow-up period was 25.6 months. The mean age of the patients was 69.4 years, and 938 (48%) of them were men. The total number of patients with HFpEF was 1129. All included studies were single-center observational cohorts. Table 1 and Table 2 summarize the research designs, baseline patient traits, number of adverse events in each analysis, and cardiac MRI metrics for each included study.

Prognostic data for ECV were reported in five studies, for native T1 in three studies, and for postcontrast T1 times in just one study. Table 3 presents the results of the primary analyses (unadjusted and adjusted HR values), including the outcomes in each study. 

### 3.3. Extracellular Volume

Five studies [10,11,12,13,14] reported outcomes regarding ECV, and all of them discovered that higher ECV was associated with an increased risk of adverse events. The study by Duca et al. demonstrated that higher ECV was linked to an augmented risk of hospitalization with HF or death from cardiovascular causes in both univariate analysis (HR:1.132; 95% CI: 1.049–1.222; *p* = 0.001) and multivariate analysis (HR_adjusted_:1.099; 95% CI: 1.005–1.201; *p* = 0.038) [10]. The study by Schelbert et al. also detected that higher ECV was a strong predictor in univariate analysis (HR: 1.93/5% ECV; 95% CI: 1.50–2,50; *p* < 0.001), as well as an independent predictor in multivariate analysis (HR_adjusted_:1.52/5% ECV; 95% CI: 1.05–2.21; *p* = 0.03), for hospitalization with HF or death from cardiovascular causes in patients with HFpEF or at risk of developing HFpEF (based on elevated levels of brain natriuretic peptide) [11]. In their study, Roy et al. established that augmented ECV was an independent predictor of adverse outcomes (death and HF) in patients with HFpEF in univariate analysis (HR: 1.07; 95% CI: 1.01–1.12; *p* = 0.015) and multivariate analysis (HR_adjusted_:1.07; 95% CI: 1.00–1.13; *p* = 0.037). However, in this study, ECV was associated with adverse outcomes among CMR parameters, but failed to reach statistical significance in the combined multivariable model, which also included clinical and hemodynamic parameters [12]. The research by Kanagala et al. also demonstrated the prognostic significance of ECV in HFpEF patients for hospitalization with HF or for all-cause mortality (HR_unadjusted_:1.519; 95% CI: 1.076–2.145; *p* = 0.018) [13]. The study revealed that increased iECV (ECV indexed to body surface area) was independently associated with cardiovascular events (HR_adjusted_:1.69; 95% CI: 1.14–2.50; *p* = 0.009). The latest study by Yang et al. also demonstrated that higher ECV fraction was associated with increased risk of all-cause mortality and HF hospitalization in univariate analysis (HR: 1.98; 95% CI: 1.10–3.56; *p* = 0.02) and multivariate analysis (HR_adjusted_:1.73; 95% CI: 1.04–2.88; *p* = 0.03) [14].

**Figure 2:**
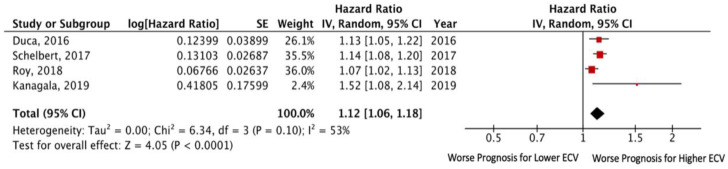
Forest plots comparing outcomes with lower and higher ECV in HFpEF patients.

The data from all studies (except for that of Yang et al. [14]) were comparable with one another due to using the same criterion for assessing the predictor (1% change), which allowed them to be used in a meta-analysis. The overall number of patients with HFpEF in these studies was 877. Endpoint death and/or HF hospitalization took place in 180 (20.5%) patients. The pooled unadjusted HR for death and/or HF hospitalization was 1.12 (95% CI: 1.06–1.18; *p* < 0.0001), without significant heterogeneity among the included studies (I^2^ = 53%, *p* = 0.10). Egger’s test yielded t = 1.63, *p* = 0.24 (Figure 2).

**Figure 3:**
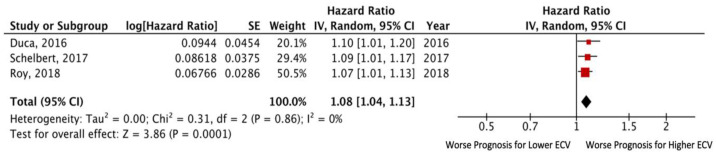
Forest plots comparing outcomes with lower and higher ECV in HFpEF patients.

Three studies reported data on adjusted HR via multivariate analysis [1,5,9]. The pooled adjusted HR for death and/or HF hospitalization was 1.08 (95% CI: 1.04– 1.13; *p* = 0.0001), without significant heterogeneity among the included studies (I^2^ = 0%, *p* = 0.86). Egger’s test yielded t = 5.77, *p* = 0.11 (Figure 3).

### 3.4. Native T1 Time

Three out of seven studies reported outcomes in relation to native T1 value. All of them found no significant association of native myocardial T1 time values with adverse cardiovascular outcomes. Duca et al. [10] and Roy et al. [12] demonstrated that native T1 times were not associated with adverse outcomes—HR: 1.005 (95% CI: 0.99–1.01; *p* = 0.103) and HR: 1.01 (95% CI: 0.99–1.01; *p* = 0.23). In a study comparing the native myocardial T1 time values of HFpEF patients versus control subjects, Kanagala et al. demonstrated that HFpEF patients had significantly higher native T1 time values (*p* = 0.021) [13]. However, the authors of that study did not report any relationships of native T1 time with clinical outcomes. Garg et al. disclosed that native T1 times were not connected to all-cause mortality (HR: 2.84; 95% CI: 0.88–6.34), and T1 mapping was the only CMR parameter associated with mortality in the HFpEF cohort, excluding amyloid cases (HR: 2.84; 95% CI 1.06–7.64) [6]. 

**Figure 4:**
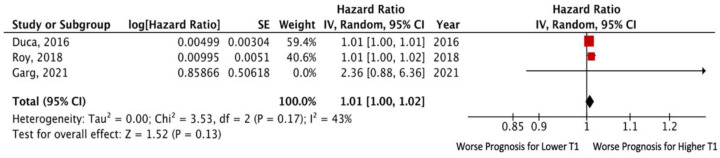
Forest plots comparing outcomes with lower and higher T1 in HFpEF patients.

Analysis of the above-mentioned studies pooled together yielded no significant association of T1 value with the risk of death or adverse cardiovascular events. The pooled unadjusted HR for death and/or HF hospitalization was 1.01 (95% CI: 1.00–1.02; *p* = 0.13), without significant heterogeneity among the included studies (I^2^ = 43%, *p* = 0.17) (Figure 4).

### 3.5. Postcontrast T1 Time

Data on the prognostic importance of postcontrast T1 time in HFpEF patients are quite limited. Only the study by Mascherbauer et al. [9] demonstrated that lower postcontrast T1 times were significantly associated with adverse cardiac events (HR: 0.99; 95% CI: 0.98–0.99). Those authors also reported that the extracellular matrix of left ventricular biopsies (*n* = 9), quantified via Tissue FAXS technology, was correlated with T1 time (r = 0.98; *p* < 0.01). 

## 4. Discussion

Due to the variability in phenotypic and clinical manifestations of HFpEF, current strategies do not fully identify all patients at high risk of HFpEF. Hence, the clinical need to identify new markers to assist with risk stratification is progressively increasing. Newer techniques, such as T1 mapping, exhibit some promise in detecting diffuse fibrosis and providing additional valuable prognostic information in HFpEF patients. Still, the use of T1 mapping for risk stratification in HFpEF patients is not widely adopted as of yet. 

This is the first meta-analysis examining the prognostic value of T1 mapping in HFpEF subjects. Its results suggest that ECV assessment can be used as an effective tool for risk stratification in patients with HFpEF. In our meta-analysis of 4 studies, involving 877 patients, with a mean follow-up period of 23.7 months, we demonstrated that higher ECV was a powerful predictor of death risk and/or hospitalization risk for HF in patients with HFpEF. However, our meta-analysis also showed that native T1 times were not associated with adverse outcomes in HFpEF patients. Data on the prognostic value of postcontrast T1 time in HFpEF patients are by all means limited; hence, future studies using such an approach to characterize the myocardium in HFpEF patients are direly needed. 

## 5. Limitations

Our study has several limitations. First, a limited number of studies were included in our analysis. Second, there is heterogeneity in the patient selection criteria and baseline characteristics between the different studies analyzed. In addition, certain groups of patients were excluded from the studies—primarily patients with hypertrophic cardiomyopathy and infiltrative heart disease. Finally, in the studies, various covariates were included in the multivariate model.

## 6. Conclusions

The higher ECV on CMR imaging in HFpEF patients is an important prognostic marker for an increased risk of death and/or HF hospitalization.

## Figures and Tables

**Table 1:** Baseline characteristics of studies included in the meta-analysis.

Study	Year	Sample Size	Study Design	Follow-Up (Months)	Magnetic Field Strength (Tesla)	Myocardial T1 Parameter	Events (*n*, %)	Outcomes
Mascherbauer [9]	2013	100 (63 *)	Prospective single-center study	22.9 ± 5.0	1.5 T	T1 time	16 (25.4%)	13 patients were hospitalized for HF; 3 patients died
Duca [10]	2016	117	Prospective single-center study	24.0	1.5 T	ECV, native T1	34 (29%)	30 patients were hospitalized for HF; 4 patients died
Schelbert [11]	2017	1174 (410 *)	Prospective single-center study	22.8	1.5 T	ECV	61 (14.9%)	19 patients were hospitalized for HF; 48 patients died; 6 did both
Roy [12]	2018	118	Prospective single-center study	11 ± 6	3.0 T	ECV Native myocardial T1 time (ms)	43 (36.4%)	32 patients were hospitalized for HF; 11 patients died
Kanagala [13]	2019	232	Prospective single-center study	48.2	3.0 T	Native myocardial T1 time (ms), postcontrast myocardial T1 time (ms), ECV	42 (18.1%)	28 patients were hospitalized for HF; 14 patients died
Yang [14]	2021	103	Retrospective single-center study	12.3	3.0 T	ECV	39 (37.9%)	39 patients reached the composite primary outcome
Garg [6]	2021	86	Retrospective single-center study	38.4	1.5 T	Native T1 values (ms)	27 (31%)	27 patients reached the endpoint of all-cause mortality

Note: ECV—extracellular volume fraction; HF—heart failure; * number of patients with HFpEF.

**Table 2:** Patient traits in the included studies.

Study	Age (Years)	Male Gender, Number (%)	LVEF	LGE Prevalence (%)	NYHA Functional Class III-IV (*n*, %)	Beta-Blockers (*n*, %)	ACE-I or ARB (*n*, %)	MRA (*n*, %)	Diuretics Other Than MRA (*n*, %)	NT-proBNP, pg/mL	LV Mass Index, g/m2	E/E‘ Ratio	LAVI, ml/m2	ECV
Mascherbauer, 2013 [9]	70 ± 7	38 (40)	64 ± 10	-	-	-	-	-	-	1343 ± 1178	59.5 ± 17.7	-	-	-
Duca, 2016 [10]	74 ± 8	36 (31)	63 ± 10	-	71 (61)	-	-	-	-	833 (396 to 1892) £	56.4 ± 13.2	-	-	29.3 ± 3.9
Schelbert, 2017 [11]	56 [44–66]	637 (54)	-	301 (25.6)	-	255 (62)	175 (43)		176 (43)	-	-	-	-	-
Roy, 2018 [12]	78 ± 8	37 (31)	64 ± 7	26 (22)	53 (45)	76 (64)	76 (64)	23 (19)	94 (80)	1747 (374 to 34,306) £	68 ± 15	18.1 ± 7.3	66 ± 29	32.9 ± 4.8
Kanagala, 2019 [13]	73 ± 8	67 (49)	56 ± 6	49 (51)	28 (29)	68 (71)	82 (85)	31 (32)	76 (79)	144 [66–250] *	51 ± 13	12.8 ± 4.8	54 ± 27	27.8 ± 4.6
Yang, 2021 [14]	58 ± 9	71 (69)	49 [39–59]			48 (46.6)	71 (68.9)	73 (70.9)	81 (78.6)	5723 [3259–8292]				36.5 [33.4–39.6]
Garg, 2021 [6]	78 ± 9	52 (61)	59 ± 12.4	-	-	-	-	-	-	-	-	-	-	-

Note: ACE-I—angiotensin-converting enzyme inhibitor; ARB—angiotensin receptor blocker; ECV—extracellular volume fraction; LVEF—left ventricular ejection fraction; LGE—late gadolinium enhancement; LAVI—left atrial volume index; MRA—mineralocorticoid receptor antagonist; £ median [min; max], * BNP, ng/L.

**Table 3:** Estimated changes in myocardial T1 parameters and corresponding HR values from Cox univariate/multivariate proportional hazard analyses.

Study	CMR Mapping Parameters	Unadjusted HR	95% CI	*p*	Adjusted HR	95% CI	*p*	Outcomes
Mascherbauer, 2013 [9]	Postcontrast T1 time (ms)	0.99	0.98–0.99	0.01	-	-	-	Hospitalization for heart failure or death from cardiovascular causes
Duca, 2016 [10]	ECV (%)	1.132	1.049–1.222	0.001	1.099	1.005–1.201	0.038	Hospitalization for heart failure or death from cardiovascular causes
	Native myocardial T1 time (ms)	1.005	0.999–1.011	0.103	-	-	-	
Schelbert, 2017 [11]	ECV (per 5% ECV increase)	1.93	1.50–2.50	< 0.001	1.52	1.05–2.21	0.03	Hospitalization for heart failure or death from cardiovascular causes
	ECV (per 1% ECV increase),	1.14	1.08–1.20	< 0.001	1.09	1.01–1.17	0.03	
Roy, 2018 [12]	ECV (%)	1.07	1.01–1.12	0.015	1.07 *	1.01–1.13	0.037	Combination of all-cause mortality or the first hospitalization for heart failure
	Native myocardial T1 time (ms)	1.01	0.99–1.01	0.23	-	-	-	
Kanagala, 2019 [13]	ECV (%)	1.519	1.076–2.145	0.018	-	-	-	Death and/or hospitalization for heart failure
	iECV (mL/m^2^)				1.69	1.107–2.113	0.01	
Yang, 2021 [14]	ECV fraction (every doubling)	1.98	1.10–3.56	0.02	1.73	1.04–2.88	0.03	All-cause mortality and heart failure hospitalization
Garg, 2021 [6]	Native myocardial T1 time (ms)	2.36	0.88–6.38	NR	-	-	-	All-cause mortality

Note: CI—confidence interval; ECV—extracellular volume fraction; HR—hazard ratio; CMR—cardiac magnetic resonance; ECV—extracellular volume fraction; iECV—extracellular volume fraction indexed to body surface area; * adjusted for CMR parameters.

## Data Availability

Data is contained within this article.
